# Psychometric properties of the Chinese version of the Quick Delay Questionnaire (C-QDQ) and ecological characteristics of reward-delay impulsivity of adults with ADHD

**DOI:** 10.1186/s12888-024-05706-2

**Published:** 2024-04-02

**Authors:** Caili Chen, Shiyu Zhang, Haiheng Hong, Sunwei Qiu, Yi Zhou, Mengjie Zhao, Meirong Pan, Feifei Si, Min Dong, Haimei Li, Yufeng Wang, Lu Liu, Edmund J. S. Sonuga-Barke, Qiujin Qian

**Affiliations:** 1 Peking University Sixth Hospital/Institute of Mental Health, 100191 Beijing, China; 2 NHC Key Laboratory of Mental Health (Peking University), National Clinical Research Center for Mental Disorders (Peking University Sixth Hospital), 100191 Beijing, China; 3 Institute of Psychiatry, Psychology & Neuroscience, King’s College London, London, UK

**Keywords:** attention-deficit/hyperactivity disorder, Delay aversion, Delay discounting, Reliability, Validity

## Abstract

**Background:**

The Quick Delay Questionnaire (QDQ) is a short questionnaire designed to assess delay-related difficulties in adults. This study aimed to examine the reliability and validity of the Chinese version of the QDQ (C-QDQ) in Chinese adults, and explore the ecological characteristics of delay-related impulsivity in Chinese adults with attention-deficit/hyperactivity disorder (ADHD).

**Methods:**

Data was collected from 302 adults, including ADHD (*n* = 209) and healthy controls (HCs) (*n* = 93). All participants completed the C-QDQ. The convergent validity, internal consistency, retest reliability and confirmatory factor analysis (CFA) of the C-QDQ were analyzed. The correlations between C-QDQ and two laboratory measures of delay-related difficulties and Barratt Impulsiveness Scale-11 (BIS-11), the comparison of C-QDQ scores between ADHD subgroups and HCs were also analyzed.

**Results:**

The Cronbach’s α of C-QDQ was between 0.83 and 0.89. The intraclass correlation coefficient of C-QDQ was between 0.80 and 0.83. The results of CFA of C-QDQ favoured the original two-factor model (delay aversion and delay discounting). Significant positive associations were found between C-QDQ scores and BIS-11 total score and performance on the laboratory measure of delay-related difficulties. Participants with ADHD had higher C-QDQ scores than HCs, and female ADHD reported higher scores on delay discounting subscale than male. ADHD-combined type (ADHD-C) reported higher scores on delay aversion subscale than ADHD-inattention type (ADHD-I).

**Conclusion:**

The C-QDQ is a valid and reliable tool to measure delay-related responses that appears to have clinical utility. It can present the delay-related impulsivity of patients with ADHD. Compared to HCs, the level of reward-delay impulsivity was higher in ADHD.

**Supplementary Information:**

The online version contains supplementary material available at 10.1186/s12888-024-05706-2.

## Introduction

Attention-deficit/hyperactivity disorder (ADHD) is a heterogeneous disorder associated with multiple neuropsychological deficits, such as executive deficits, delay-related difficulties (e.g., excessive temporal discounting, delay aversion), and deficits in temporal processing [1–4]. These ADHD symptoms are dissociable, with substantial subgroups of patients affected in only one domain [5, 6]. The preference for smaller, sooner (SS) over larger, later (LL) rewards, reward-delay impulsivity, is one of the strongest motivational markers in ADHD [7–11]. This tendency is found across development, including in adulthood [9, 12, 13]. Such a pattern of choice between rewards is related to a range of negative outcomes, including criminality, substance use, and greater functional impairment, such as in time and money management [14] as well as in learning and study strategies [15].

Sonuga-Barke suggested that this tendency to choose SS over LL rewards in ADHD is driven by two components: steeper temporal discounting of the future reward and acquired delay aversion (a negative emotional reaction to the imposition of delay) [16]. Laboratory studies support a role for both exaggerated temporal discounting and delay aversion. In terms of delay aversion, ADHD individuals show excessive alerting to delay-related cues, which is similar to that of anxious children in the face of physical and social threats [17]. They also display higher levels of delay-related frustration during long, boring tasks [18, 19]. Neuroimaging studies have demonstrated delay aversion in ADHD, with increased involvement of emotional brain regions (i.e., the amygdala) in response to delay-related cues [20, 21]. In terms of temporal discounting, many studies using real-time and hypothetical tasks have shown that individuals with ADHD discount the value of future rewards compared to controls [9, 10]. The important roles of both ADHD components are supported by experimental studies that manipulated prereward delay and total delay independently. In a study by Marco et al. (2009), ADHD individuals exhibited similar preference for the SS reward and LL reward when trial length (i.e., total delay) was the same for both options and increased preference for the SS reward when that choice was linked to the reduction of delay by removing the postreward delay period [8].

Measurements of temporal discounting and delay aversion typically involve laboratory tasks, most of which use paradigms that involve the choice between SS and LL rewards, specifically, the simple choice paradigm (SCP) and the temporal discounting paradigm (TDP). The delay parameters in these tasks may need to be extended to very high levels to assess performance in adulthood, given adult tolerance for long periods of delay. As a result, Clare et al. (2010) developed a short questionnaire, the Quick Delay Questionnaire (QDQ), to yield separate estimates of the two constructs in adults [22]. The QDQ contains two subscales: delay aversion and delay discounting. The reliability and validity of the QDQ have been verified. In 2017, Thorell and colleagues developed a Swedish version of the scale and further verified the reliability and validity of this scale in a sample that included both patients with ADHD and clinical and nonclinical controls [14]. In addition, the QDQ was found to be mainly related to hyperactivity and impulsivity symptoms rather than inattention symptoms. There was a significant association of QDQ scales with impairments in activities of daily living. The QDQ has been adapted into several other versions to assess delay-related difficulties in children with ADHD [23, 24].

Some studies have also found that there may be differences in the level of reward-delay impulsivity in different ADHD subtypes and genders [25, 26]. Scheres et al.‘s study reported that steep temporal reward discounting was observed in children and adolescents with ADHD-C but not in those with ADHD-I [26]. Other studies have shown that girls with ADHD show a greater delay discounting than boys with ADHD [27, 28]. A recent meta-analysis explored gender differences in the performance of two paradigms measuring the reward-delay impulsivity between normally developing healthy people and ADHD, and also showed that female ADHD were more inclined to choose SS rewards than male ADHD, showing greater reward-delay impulsivity [25]. However, it is unclear whether there are also gender, subtype differences in reward-delay impulsivity levels in adults with ADHD.

At present, there are few studies on ADHD delay-related behaviours in China. Studies have mainly focused on children [29–31]. The research questions of this study are whether QDQ is also applicable to the Chinese population, and what are the characteristics of the reward-delay impulsivity in Chinese adults with ADHD? Our research hypotheses are that QDQ adult self-assessment is well adapted to Chinese cultural background; adult ADHD may have abnormal reward-delay impulsivity, and ADHD subgroups may have different reward-delay impulsivity levels. The aim of this study was to develop a Chinese version of the QDQ (C-QDQ) and verify the reliability and validity of the C-QDQ in the Chinese population, and explore the ecological characteristics of reward-delay impulsivity in Chinese adults with ADHD. The novelty of this study is that it will be the first time to introduce QDQ scale in China to make up for the lack of self-assessment tools for adult reward-delay impulsivity in China. More specifically, we aimed to:

(1) Verify the reliability and validity of the C-QDQ (i.e., internal consistency and test-retest reliability as well as content validity, construct validity, and convergent validity).

(2) Explore the differences of reward-delay impulsivity between ADHD and healthy controls, as well as ADHD groups with different sex and subtypes.

## Methods

### Participants

A total of 302 participants were recruited. Adults with ADHD were recruited from the outpatient clinic of Peking University Sixth Hospital/Institute of Mental Health, China. Healthy controls were recruited through advertisements in communities in China. The inclusion criteria for participants with ADHD were as follows: (1) met the diagnostic criteria for ADHD according to the American Diagnostic and Statistical Manual of Mental Disorders, 5th edition (DSM-5) [32]; and (2) aged above 18 years. The assessment used the Conners’ Adult ADHD Diagnostic Interview for the DSM-IV (CAADID) [33], which assesses both current and childhood symptoms of ADHD. To obtain DSM-5 diagnoses of ADHD, the DSM-IV criteria was converted to DSM-5 criteria. This diagnostic criterion is available, similar conversions have been adopted in other studies [34, 35]. In addition to ADHD diagnosis, some participants in the ADHD group also met the DSM–IV criteria for comorbid mental disorders, which were assessed using the Structured Clinical Interview for DSM-IV Axis 1 Disorders (SCID-I) [36]. The healthy controls did not meet the criteria for a diagnosis of ADHD based on an evaluation by trained psychiatrists.

This study was approved by the Ethics Committee of the Peking University Sixth Hospital/Institute of Mental Health, China ((2022) Ethics review number (41)). All participants consented to participate and signed an informed consent form.

### Measures

#### The Quick Delay Questionnaire (QDQ)

is a self-rating scale used to measure delay-related behaviours in adults, and all items are presented in the original publication [22]. It contains 10 items that relate to reactions and attitudes to delay-related activities and situations in adult daily life, and the first 4 items are reverse scored. Items are rated on a five-point Likert scale ranging from 1 (“not like me at all”) to 5 (“very like me”), and higher scores indicate greater delay aversion. The scale is divided into two subscales: delay aversion and delay discounting. The internal consistency of this scale is good, Cronbach’s alpha values of the two subscales were 0.77 and 0.68, respectively, and test-retest performance of the two subscales was also good (*r* = 0.80 for delay aversion, *r* = 0.81 for delay discounting) [22].

#### Barratt Impulsiveness Scale-11 (BIS-11)

The scale consists of 30 items scored on a 4-point scale, including attentional, nonplanning, and motor subscales.The higher the score is, the more impulsive the individual is [37]. The psychometric properties of its Chinese version were verified, the Cronbach’s a coefficients of the total BIS-11 and its subscale scores were all good (a = 0.67–0.89), it is a reliable instrument for assessing individual impulsivity [38].

#### Two-Choice Impulsivity Paradigm (TCIP)

The TCIP (a modified version of the task reported in [39], see also [40]) is a nonverbal computer-based task that measures participant preference for SS or LL rewards. Use of this task has been verified in a series of adult samples [41]. In the TCIP, participants choose between waiting for five seconds to get five points (a circle) or waiting for 15 s to get 15 points (a square), with the goal of earning as many points as possible. The dependent variable for this task is the percentage of SS rewards chosen. The more individuals prefer SS rewards, the more delay aversion they exhibit.

#### Delay Discounting Task (DDT)

This task refers to the design of a task in a Chinese study [42, 43], which is similar to that in [44]. The task requires participants to choose between a fixed SS reward (50 yuan for 0 days) and an LL reward with a variable delay. Participants are told that the rewards and delays in the experiment are hypothetical and that they will not actually receive the rewards. However, they are instructed to consider the scenario as if it was real when making a decision. Finally, the value of the delayed discount rate (k) is calculated as the impulsivity index. The higher the value of k is, the greater the delay discounting.

### Procedure

Because this study was conducted in China, we first obtained translation permission from the original author via email. Then, the questionnaire was translated and back-translated according to Brislin’s translation procedure. It should be noted that translation can have a significant impact on a scale. However, because the items of the QDQ are very clear, the C-QDQ was generated without any problems. Participants completed all questionnaires carefully. Those adults with ADHD and healthy controls who willing to participate in the task also completed two neuropsychological tasks (TCIP and DDT) after completing the questionnaire; these tasks were performed in a specific quiet room in the hospital. Referring to the interval of the original scale study [22], 2 weeks later after completing the questionnaire, partial participants with ADHD completed the C-QDQ a second time. Within one year of data collection, we randomly selected one month to send retest questionnaires to participants with ADHD, who completed the C-QDQ for the first time exactly two weeks, and finally collected 42 questionnaires.

### Data preprocessing

Calculation of the k value of the DDT task. Trials with missing choice data were excluded from processing. Trials with response times (RTs) < 100 ms were also excluded. The other trials were defined as hit trials and were included in the analyses [43, 45]. First, a logistic function was used to calculate the indifference point of each delay, the time at which there was an equal probability of selecting the immediate versus the delayed option at the specific delay. Then, the discounted value (DV) for each delay was calculated with the following formula: DV = 50/indifference point. DVs were fit against the delays with a hyperbolic function called the discounting curve [46]: DV = 1/(1 + kD), where D is the length of the delay, and k is an individual’s discounting rate. Larger k values indicate that the delayed rewards are discounted more steeply and, consequently, that the participant is more impulsive. Before analysis, the discounting parameter (k) was normalized by a logarithmic transformation [47].

### Statistical analysis

Descriptive statistics were used to summarize the demographic information of the sample. AMOS (version 25.0; IBM) was applied to conduct the confirmatory factor analysis (CFA). Other data analyses were conducted using SPSS (version 25; IBM).

Cronbach’s α coefficients were calculated to assess the internal consistency of the C-QDQ total and subscale scores, and intraclass correlation coefficients (ICCs) were calculated to assess test-retest reliability. Content validity was evaluated using the content validity index (CVI), which mainly includes item-level content validity (I-CVI) and average scale-level content validity (S-CVI/AVE). The expert panel was asked to score each item regarding the relevance to the total questionnaire on a 4-point scale (1: very irrelevant; 4: very relevant). I-CVI was defined as the number of experts with a score of 3 or 4 for each item/total number of experts (I-CVI ≥ 0.78 is good). S-CVI/AVE was defined as the number of occurrences of score 3 or 4 in all items/the total number of ratings (S-CVI/AVE ≥ 0.9 is good) [48]. Construct validity was verified by CFA, which was performed using robust maximum likelihood estimation. Goodness of fit was examined by values of the χ^2^/df (ratio of chi-square and degrees of freedom), root mean square error of approximation (RMSEA), standardized root mean squared residual (SRMR), comparative fit index (CFI), incremental fit index (IFI) and Tucker‒Lewis index (TLI). A model with 1 < χ^2^/df < 3.0; RMSEA < 0.08; SRMR < 0.08; and CFI, IFI and TLI > 0.90 indicates an adequate fit [49, 50]. Moreover, modification indices were examined to determine opportunities to improve model fit if it was not ideal.

The study also tested the convergent validity of the scale by partial correlation analysis, which examined correlations of the C-QDQ subscores with the BIS-11 total score, the delay discounting rate of the DDT and the preference for SS rewards of the TCIP.

Explore the ecological characteristics of reward-delay impulsivity in Chinese adults with ADHD by comparing the C-QDQ scores between groups. If C-QDQ score, age, years of education and other quantitative data conform to normal distribution, independent sample t-test was used for comparison of two groups, and analysis of variance (ANOVA) was used for comparison of multiple groups. Wilcoxon rank sum test was used for comparisons that did not conform to normal distribution, Kruskal-Wallis test was used for comparisons between more than two groups. Bonferroni correction was used as the post hoc test. Pearson χ2 test was used to compare gender and other categorical variables among all groups. When comparing differences in C-QDQ scores between groups, general demographic data for differences between groups were present as covariates. In the gender subgroup analysis, two-factor factorial design ANOVA was used to analyse the main effects of diagnosing gender, the interaction between the two factors and simple effects. We conducted a normality test on the data, and found that C-QDQ scores and years of education were in line with normal distribution, while age was not. See Supplementary Materials for details.

## Results

### General demographic data

The study involved 302 adults, including 209 adults with ADHD and 93 healthy controls. The median age of the total sample was 25 (23, 29) years, and 46.7% of participants were male. The average number of years of education was 16.88 ± 2.56. Descriptive data and group differences with regard to demographic variables are presented in Table 1. No significant group differences were found for age or gender, but the groups differed significantly with regard to educational level, the ADHD group had fewer years of education than the healthy controls group.

**Table 1:** The demographics of all participants

	ADHD (*n* = 209)	HCs (*n* = 93)	*p*
Gender [n (%men)]	100 (47.8%)	41 (44.1%)	0.545
Age (years)	26 (23, 30) ^a^	25 (24, 26) ^a^	0.065
Educational (years)	16.34 ± 2.56 ^b^	18.08 ± 2.14 ^b^	< 0.001***
Subgroup [n (%)]			
ADHD-I	117 (56%)	-	-
ADHD-C	92 (44%)	-	-

Note: a: [median (P25, P75)]; b: means ± standard deviation; ADHD: attention-deficit/hyperactivity disorder group; ADHD-I: ADHD-predominantly inattention type; ADHD-C: ADHD-combined type; HCs: healthy controls; ***: *p* < 0.001

In the ADHD group, 56% of participants were diagnosed with ADHD-inattention type (ADHD-I), 44% with ADHD-combined type (ADHD-C). 67% of participants had comorbid diagnoses: major depressive disorder (42.5%), bipolar disorder (14.8%), dysthymia (2.4%), anxiety disorders (26.8%) [including generalized anxiety disorder (6.7%), social anxiety disorder (11.9%), specific phobia (4.8%), panic disorder (5.3%), agoraphobia (1.4%), and unspecified anxiety disorder (2.4%)], obsessive-compulsive disorder (10%), eating disorder (5.3%), and alcohol use disorder (2.4%). Sixty-three participants had more than one diagnosis.

### Reliability of the C-QDQ

#### Internal consistency reliability

The Cronbach’s α coefficient of the full scale of the C-QDQ was 0.89, and the Cronbach’s α coefficients of the delay aversion and delay discounting subscales were 0.87 and 0.83, when including all participants. It indicated that the items of the scale had high internal consistency. Similar results were found when studying internal consistency separately for the ADHD sample and healthy sample (αs ranging between 0.73 and 0.85).

#### Test-retest reliability

Forty-two randomly chosen patients with ADHD completed the C-QDQ twice (separated by an interval of 2 weeks) to evaluate the test–retest reliability. The results showed that the ICC of the total scores was 0.83 (*P* < 0.001), and the ICC values of the delay aversion and delay discount subscales were both 0.80 (*P* < 0.001). The test–retest correlation coefficient of the C-QDQ showed that the stability of the C-QDQ was high.

### Validity of the C-QDQ

#### Content validity

Six committee experts were invited to review the contents of the scale and score each item regarding its relevance to the total questionnaire. All the experts gave a score of 3–4 for each item. Therefore, both the I-CVI and S-CVI/AVE values of the C-QDQ were 1.0, indicating adequate content validity.

#### Construct validity

**Fig. 1:**
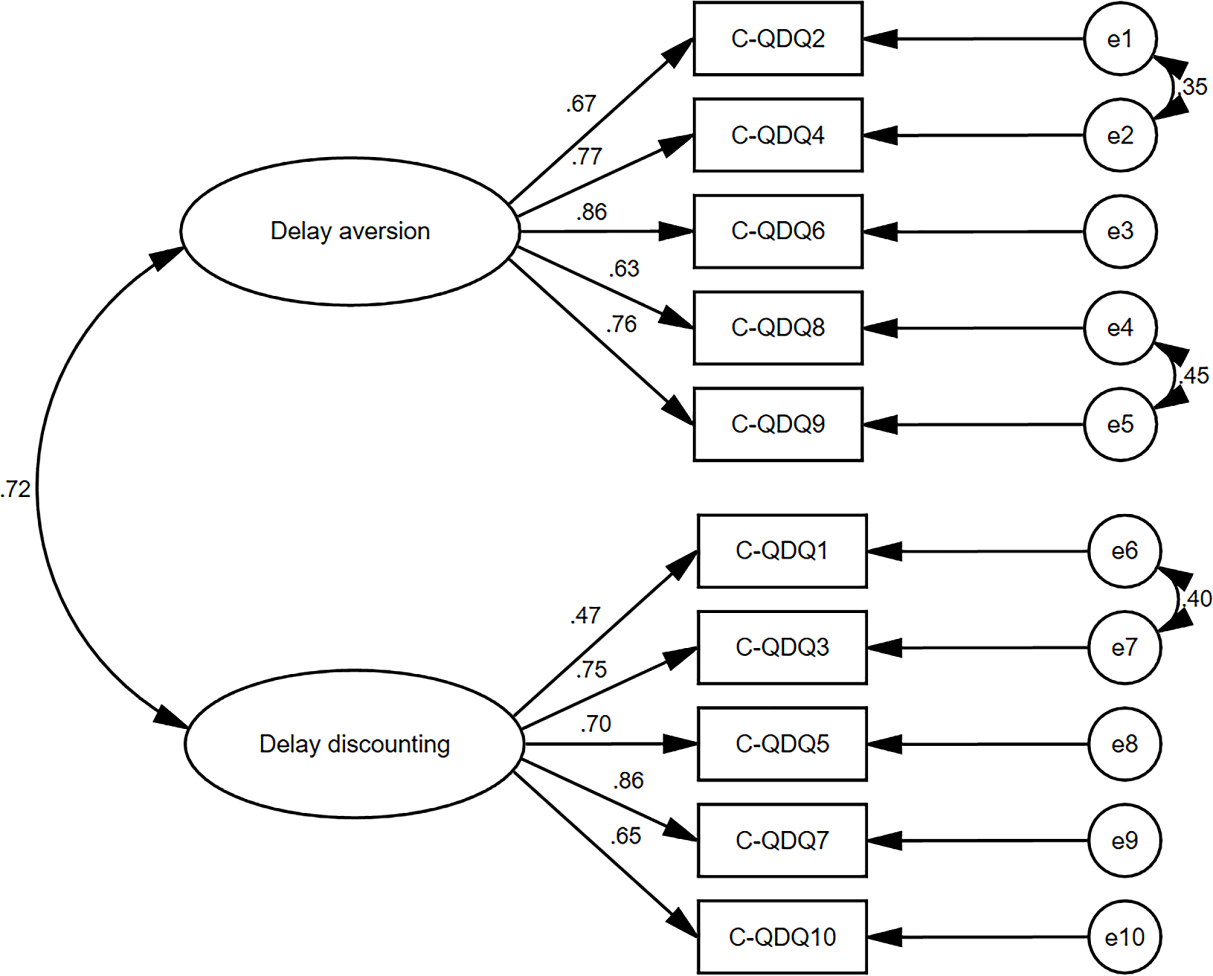
Structural equation model, CFA

The original scale had a two-factor structure, namely, factors of delay aversion and delay discounting. CFA was performed to evaluate the two-factor model fit of the C-QDQ, with the ten items as observed variables and the two factors as latent variables. Most factor loadings were satisfactorily high (0.47–0.86; Fig. 1). However, two latent variables were highly correlated (*r* = 0.72, *p* < 0.01) (see Fig. 1). The initial fit of the model was insufficient, CFI = 0.887, RMSEA = 0.131, χ2/df = 6.131. According to the modification indices and the specific content of the items, three sets of items can be modeled in association to improve model fit. Both item 1 and item 3 are attitudes toward long-term benefits (item 1: “Even if I have to wait a long time for something I won’t give up if it is important to me”; Item 3: “I will often chooses a task because it is beneficial in the long term even if it doesn’t offer immediate reward”). Both item 2 and item 4 related to positive attitudes of queuing/waiting (item 2: “I am usually calm when I have to wait in queues”, item 4: “I feel relaxed when waiting for things”). Item 8 and item 9 are negative emotional reactions to waiting (item 8: “I feel frustrated when I have to wait for someone else to be ready before I can do something”; Item 9: “Having to wait for things makes me feel stressed and tense”). After the above modification, the goodness-of-fit assessment showed χ^2^/df = 2.741, RMSEA = 0.076, SRMR = 0.040, CFI = 0.965, IFI = 0.965, and TLI = 0.949. The CFA model fit indices before and after modification are described in Table 2.

**Table 2:** Model fit indices of the CFA before and after modification

Models	χ2/df	RMSEA	SRMR	CFI	IFI	TLI
before modification	6.131	0.131	0.060	0.887	0.888	0.851
after modification	2.741	0.076	0.040	0.965	0.965	0.949

Note: CFA = Confirmatory factor analysis; χ2/df: ratio of chi-square and degrees of freedom; RMSEA: root mean square error of approximation; SRMR: standardized root mean square residual; CFI: Comparative fit index; IFI: Incremental fit index; TLI: Tucker-Lewis index

#### Convergent validity

As described in the Methods section, the convergent validity of the C-QDQ was examined by exploring the association of the C-QDQ subscale scores with the BIS-11 total score and performance on the two neuropsychological tasks (see Table 3). For all participants, both C-QDQ subscale scores were significantly correlated with the BIS-11 total score (*r* = 0.380 for delay aversion, *p* < 0.001; *r* = 0.495 for delay discounting, *p* < 0.001). C-QDQ subscale scores were not significantly associated with performance on the DDT (*r* = 0.040 for delay aversion, *p* > 0.05; *r* = -0.124 for delay discounting, *p* > 0.05). C-QDQ subscale scores were significantly associated with performance on the TCIP (*r* = 0.219 for delay aversion, *p* < 0.01; *r* = 0.356 for delay discounting, *p* < 0.001). But, the significance of the correlation between C-QDQ subscale scores and the performance on the TCIP disappeared when evaluating the convergent validity of C-QDQ separately for the healthy controls (see Table S1 in Supplementary Materials), which may be related to the small sample of healthy controls. Results similar with the total sample were found when evaluating the convergent validity of C-QDQ separately for the participants with ADHD (see Table S2 in Supplementary Materials).

**Table 3:** The correlations between C-QDQ and BIS-11/DDT/TCIP (*n* = 302)

	C-QDQ	
	Delay aversion	Delay discounting
BIS-11	0.380***	0.495***
DDT^#^	0.040	-0.124
TCIP^^^	0.219**	0.356***

Note: ^#^=192; ^=172; **p < 0.01; ***p < 0.001. C-QDQ: the Chinese version of the Quick Delay Questionnaire; BIS-11: Barratt impulsiveness scale-11; DDT: delay discounting rate in delay discounting task; TCIP: the percentage of smaller sooner reward in two-choice impulsivity paradigm

#### Difference in reward-delay impulsivity level between groups

Compared with the healthy controls, the ADHD scored significantly higher (*p* < 0.001) on both subscales of the C-QDQ (see Table 4). The ANOVA results of diagnosis and gender on the C-QDQ scores showed that the main effect of diagnosis was significant, and the interaction effect between diagnosis and gender was partially significant (see Table 5). In the ADHD group, the simple effect of gender on C-QDQ total score and delay discounting subscale score were significant (F = 4.42, *P* = 0.036; F = 4.227, *P* = 0.041), but not on the delay aversion subscale score (F = 1.33, *P* = 0.250). In healthy controls group, the simple effects of gender on C-QDQ total score and two subscales score were not significant (all *p* > 0.05). In terms of QDQ delay discounting subscale score, female ADHD were higher than male ADHD (*p* < 0.05; see Table 6); there was no significant difference between healthy females and males (*p* > 0.05; see Table 6).

**Table 4:** Comparison of C-QDQ scores between ADHD and healthy controls

	ADHD (*n* = 209)	HCs (*n* = 93)	*p*
C-QDQ total scores	31.48 ± 6.49	20.41 ± 5.42	< 0.001***
Delay aversion	17.02 ± 4.08	10.97 ± 3.64	< 0.001***
Delay discounting	14.46 ± 3.87	9.44 ± 2.52	< 0.001***

Note: C-QDQ: the Chinese version of the Quick Delay Questionnaire; HCs: healthy controls; ***: *p* < 0.001

**Table 5:** Analysis of variance between diagnosis and gender on C-QDQ scores

		Male		Female	Diagnosis		Gender		Diagnosis* Gender	
					F	*P*	F	*P*	F	*P*
C-QDQ total scores	ADHD		30.55 ± 6.04	32.34 ± 6.78	203.28	< 0.001***	0.38	0.538	3.08	0.080
	HCs		20.88 ± 5.49	20.04 ± 5.39						
Delay aversion	ADHD		16.69 ± 3.76	17.32 ± 4.34	147.83	< 0.001***	0.10	0.753	0.98	0.324
	HCs		11.15 ± 3.71	10.83 ± 3.61						
Delay discounting	ADHD		13.86 ± 3.82	15.02 ± 3.86	133.37	< 0.001***	0.15	0.700	3.89	0.049*
	HCs		9.73 ± 2.52	9.21 ± 2.52						

Note: C-QDQ: the Chinese version of the Quick Delay Questionnaire; HCs: healthy controls; *: *p* < 0.05, **: *p* < 0.01, ***: *p* < 0.001.Two-factor factorial covariance analysis was used to compare C-QDQ scores, with age and years of education as covariables

**Table 6:** Demographic and delay discounting level of ADHD and healthy controls with different genders

	ADHD		HCs		Group comparisons	
	Men (*n* = 100)	Women (*n* = 109)	Men (*n* = 41)	Women (*n* = 52)	ADHD men vs. women *p*	Health control men vs. women *p*
Age (years)	27 (23, 31) ^a^	26 (22, 29) ^a^	25 (23, 26) ^a^	25 (24, 27) ^a^	0.152	0.625
Educational (years)	16.08 ± 2.83 ^b^	16.59 ± 2.27 ^b^	17.68 ± 3.64 ^b^	18.38 ± 2.12 ^b^	0.118	0.153
Delay discounting	13.86 ± 3.82 ^b^	15.02 ± 3.86 ^b^	9.73 ± 2.52 ^b^	9.21 ± 2.52 ^b^	0.015*	0.467

Note: a: [median (P25, P75)]; b: means ± standard deviation; HCs: healthy controls; *: *p* < 0.05

Descriptive data and ADHD subgroups differences with regard to demographic variables are presented in Table 7. No significant group differences were found for age or gender, but the groups differed significantly with regard to educational level and C-QDQ scores. The education level of healthy controls group were higher than ADHD subgroups (all *p* < 0.05), but there was no significant difference between ADHD-I group and ADHD-C group (*p* > 0.05). The C-QDQ total score and delay aversion subscale score in ADHD-C group were higher than ADHD-I group, but the lowest in healthy controls group (all *p* < 0.05). The delay discounting subscale score in ADHD-I group and ADHD-C group were higher than healthy controls (all *p* < 0.05), but there was no significant difference between ADHD-I group and ADHD-C group (*p* > 0.05).

**Table 7:** Comparison of demographic data and C-QDQ scores between ADHD subgroups and healthy controls. Note: a: [median (P25, P75)]; b: means ± standard deviation; ADHD-I: ADHD-predominantly inattention type; ADHD-C: ADHD-combined type; HCs: healthy controls; ***: *p* < 0.001

	ADHD-I (*n* = 117) Group(1)	ADHD-C (*n* = 92) Group(2)	HCs (*n* = 93) Group(3)	*p*	Post hoc
Gender [n (%men)]	51 (43.6%)	49 (53.3%)	41 (44.1%)	0.316	-
Age (years)	26 (22, 31) ^a^	26 (23, 30) ^a^	25 (24, 26) ^a^	0.182	-
Educational (years)	16.42 ± 2.82 ^b^	16.25 ± 2.20 ^b^	18.08 ± 2.14 ^b^	< 0.001***	1, 2 < 3
C-QDQ total scores	30.29 ± 6.52 ^b^	33.00 ± 6.15 ^b^	20.41 ± 5.42 ^b^	< 0.001***	2 > 1 > 3
Delay aversion	15.86 ± 4.05 ^b^	18.49 ± 3.62 ^b^	10.97 ± 3.64 ^b^	< 0.001***	2 > 1 > 3
Delay discounting	14.43 ± 3.94 ^b^	14.51 ± 3.80 ^b^	9.44 ± 2.52 ^b^	< 0.001***	1, 2 > 3

The demographic data and C-QDQ scores of the ADHD group with or without comorbidity and the healthy controls group are shown in Table 8. No significant group differences were found for age or gender, but the groups differed significantly with regard to educational level and C-QDQ scores. The education level of healthy controls group was higher than ADHD group with or without comorbidity (all *p* < 0.05), but there was no significant difference between ADHD groups with and without comorbidity (*p* > 0.05). The C-QDQ total score and delay discounting subscale score in ADHD group with comorbidity were higher than ADHD group without comorbidity, but the lowest in healthy controls group (all *p* < 0.05). There was no significant difference in delay aversion scale score between ADHD groups with or without comorbidity (*p* > 0.05), but both groups were higher than healthy controls (all *p* < 0.05).

**Table 8:** Demographic data and C-QDQ scores between ADHD groups with or without comorbidity and healthy controls

	ADHD without comorbidity (*n* = 69) Group(1)	ADHD with comorbidity (*n* = 140) Group(2)	HCs (*n* = 93) Group(3)	*p*	Post hoc
Gender [n (%men)]	41 (59.4%)	59 (42.1%)	41 (44.1%)	0.052	-
Age (years)	27 (22, 31) ^a^	26 (23, 30) ^a^	25 (24, 26) ^a^	0.152	-
Educational (years)	16.41 ± 2.49 ^b^	16.31 ± 2.60 ^b^	18.08 ± 2.14 ^b^	< 0.001***	1, 2 < 3
C-QDQ total scores	29.81 ± 6.72 ^b^	32.31 ± 6.23 ^b^	20.41 ± 5.42 ^b^	< 0.001***	2 > 1 > 3
Delay aversion	16.20 ± 4.10 ^b^	17.42 ± 4.02^b^	10.97 ± 3.64 ^b^	< 0.001***	1, 2 > 3
Delay discounting	13.61 ± 3.67 ^b^	14.89 ± 3.92 ^b^	9.44 ± 2.52 ^b^	< 0.001***	2 > 1 > 3

Note: a: [median (P25, P75)]; b: means ± standard deviation; HCs: healthy controls; ***: *p* < 0.001

## Discussion

The presented study translated and examined the validity and reliability of the C-QDQ, a rating tool used to measure adult delay-related behaviours. The data were collected from participants with ADHD and healthy controls. The comprehensive evaluation showed that this scale has satisfactory reliability and validity.

In terms of internal consistency, Cronbach’s α coefficient between 0.70 and 0.90 is ideal, with exceeding lower bound meaning a low reliability, and exceeding higher bound meaning too many similar items [51, 52]. In terms of test-retest reliability, a coefficient of 0.75 indicates sufficient retest reliability [53]. In Clare et al.‘s study, the test-retest coefficients of the total QDQ and the two subscale scores were all above 0.8, and the internal consistency was satisfactory [22]. In Thorell and colleagues’ study, the internal consistency of the QDQ was found to be adequate for scores on the two QDQ subscales, whether for all participants or separately for the clinical and nonclinical sample (Cronbach’s αs ranging between 0.71–0.83) [14].In the present study, both the internal consistency coefficient and test-retest coefficient of the C-QDQ and the two subscales were good. It is worth noting that in Clare et al.‘s study, the sample included was the general adult population recruited by the network platform, and no individuals were excluded from the sample for any reason, their participants with an average age of 23.81 years old, range 18–77 years old and 86% women [22]. In Thorell and colleagues’ study, the sample included not only patients diagnosed with ADHD, health controls, but also patients diagnosed with clinical disorders other than ADHD [14]. In our study, the sample mainly included ADHD and health controls, the median age of the total sample was 25 years, and 46.7% of participants were male. However, all studies recruited adults and calculated internal consistency including all participants, and the results were good, indicating that QDQ always has good reliability in the adult population.

For the content validity of the scale, it was evaluated by six mental illness experts, and it showed good content validity at both the scale level and item level. This finding is in line with the expectation of using this scale to measure delay-related behaviours in this study.

The CFA verified the two-factor structure of the original scale, and the results showed that the factor structure model fit the C-QDQ data well. Therefore, the scale maintained a good scale structure even in a different language. But it’s worth noting, the two C-QDQ subscales respectively evaluate different aspects of delay reward, while this study found a high correlation between the two subscales. The reason may be that delay aversion reflects the negative experience of waiting [16], while delay discounting represents the decision-making attitude towards future reward, which themselves include waiting. Admittedly, this may also indicate that the discriminant validity of the C-QDQ is inadequate. In addition, we improved the model fit according to the modification indices based on the correlation between items. Although the correlation between items is not difficult to understand from the perspective of item content, but it may also indicate inadequate discriminant validity of the scale.

In terms of the convergent validity of the C-QDQ, according to previous research, individuals with high impulsivity are more inclined to choose SS rewards [54]. We believe that both delay aversion and delay discounting are related to the BIS-11 total score, which reflects the overall impulsivity characteristics of individuals [38]. As expected, both C-QDQ subscale scores were significantly positively correlated with the BIS-11 total score, indicating that people with higher trait impulsivity are more likely to show more prominent delay-related difficulties, including a higher delay discounting rate and greater delay aversion.

In terms of the correlation between the C-QDQ scores and two laboratory measures of delay-related difficulties, the present study found that the TCIP, which is regarded as a tool to measure delay aversion, was correlated not only with the delay aversion score but also with the delay discounting score. As a tool to measure delay discounting, the DDT was not significantly related to the delay discounting score and was not significantly related to the delay aversion score. The C-QDQ appeared to be somewhat contradictory in its correlation with the two laboratory tasks, but this may be due to structural differences in the measurement of different neuropsychological paradigms [55]. First, the TCIP repeats the same choice, which may lead to greater boredom than the DDT task. Second, there is a true delay in the TCIP, as participants can only proceed to the next option after the delay period is over. According to the delay aversion hypothesis, individuals with ADHD will experience more negative emotions when they have to wait, prompting them to choose SS rewards [56]. Previous studies have found a more pronounced preference for SS rewards related to actual delay experience and longer delay [29, 30]. In this study, the proportion of SS choices in the TCIP was calculated to reflect participants’ negative emotions in the face of delay. However, it is worth noting that the participants may choose SS rewards for reasons other than emotions. For example, individuals may choose SS rewards because they are not interested in the experiment or because they are eager to end the experiment for personal reasons. TCIP performance is likely influenced by factors other than the delay-related negative emotions, while the delay aversion subscale measures only the delay-related negative emotions, which made the conclusion of this study more conservative. Therefore, there was a significant correlation between the delay aversion score and TCIP performance, which supported the role of delay-related negative emotions on impulsive choice.

TCIP performance was also associated with the delay discounting score, as the TCIP may essentially be a delay discounting task, even though it is usually thought to reflect delay aversion. In fact, a fixed delay discounting rate (k) is set in advance in the TCIP. If the participant has a 50% choice rate for SS rewards, it means that the participant has a discounting rate of k; if the percentage of SS rewards is greater than 50%, the discounting rate is higher than k. Delay discounting is the depreciation of the value of the future delayed reward, and in response to the choice, the participant will choose more SS rewards, which explains why the TCIP was also related to the delay discounting score. Therefore, the correlations between the C-QDQ subscales and TCIP may prove the ideal convergent validity not only of the delay aversion subscale, but also of the delay discounting subscale.

However, DDT performance was not related to C-QDQ subscale scores, which may be partly because the DDT does not include an actual waiting period, and delay aversion may not be as obvious if the delay is only imagined. Imagined delay aversion and discounting of the delayed reward may be more easily compensated by the adult’s past experience of successfully enduring the delay and receiving the rewards. In addition, previous studies comparing laboratory and self-assessment measures of executive function, which are based on independent but related mental structures [57–60], found that the C-QDQ score is associated with TCIP performance but not with DDT performance, which may also indicate that the structure of the C-QDQ overlaps more with that of the TCIP. Although the current results cannot indicate superiority or inferiority of the self-rating scale versus the laboratory task, the combination of the two in future studies may address concerns that the delay-related difficulties are not fully captured using the laboratory task alone [14, 61].

By comparing the difference of C-QDQ scores between adult ADHD patients and healthy controls, we explored the ecological characteristics of reward-delay impulsivity of adults with ADHD: The level of reward-delay impulsivity in ADHD group and all subgroups was higher than healthy controls. The level of delay aversion in ADHD-C group was higher than ADHD-I group. The level of delay discounting in female ADHD was higher than male ADHD. Specifically, adults with ADHD scored significantly higher on both two QDQ subscales than healthy controls, excluding possible effects of gender, age, and the level of education.

This study found that there was an interactive effect between diagnosis and gender. Only in the ADHD group, gender has a significant impact on the C-QDQ total score and delay discounting subscale score. The delay discounting subscale score of female ADHD was significantly higher than male ADHD, which was consistent with the results of most previous studies [25, 27, 28]. Patros et al.’s study found that girls and boys with ADHD have different patterns of cognitive control and delay discounting, girls with ADHD (but not boys) show an increase in delay discounting in real delay discounting tasks, they were more likely to choose immediate small rewards and showed greater reward-delay impulsivity [28]. However, previous evidence for gender differences in delay discounting levels comes mainly from children, so the current study adds to the evidence for gender differences in delay discounting in adults with ADHD. This study did not find gender differences in the level of delay aversion within the ADHD group, suggesting that both male and female ADHD experience the same level of negative emotions when faced with delay. There have been no previous studies on this, it needs to be further verified in future studies.

In addition, the study also analysed the differences in C-QDQ scores between different subgroups of ADHD patients and healthy controls. The study found that compared with the ADHD-I group, the ADHD-C group had more obvious negative emotions towards delay, but the degree of discounting for future rewards was basically the same as the ADHD-I group. However, both two ADHD subgroups were more averse to delay than the healthy controls, and the degree of discounting for future rewards was greater. This is different from the results of Scheres et al., who showed that compared with ADHD-I and healthy children and adolescents, children and adolescents with ADHD-C showed greater delay discounting, and there was no inter-group difference in the subjective score of waiting difficulty [62]. The results of this study may be different from those of Scheres et al. due to the following differences: (1) The subjects of Scheres et al. ‘s study were children but not adults; (2) Delay discounting was measured in their study using a delay discounting task, while the possibility that the scale is different from the paradigm has been discussed above; (3) The difficulty in waiting scale, which mainly evaluates the difficulty of experiencing a wait during a delay discounting task, may be different from the focus of delay-related negative emotions reflected in the C-QDQ Delay aversion subscale. However, because there are few studies on the differences in reward-delay impulsivity between different subtypes of adults with ADHD, further verification is needed in future studies.

In view of the large proportion of ADHD with comorbidity, we further compared the C-QDQ scores of the ADHD with or without comorbidity and the healthy controls to analyse the influence of comorbidity on the level of reward-delay impulsivity. Compared with ADHD without comorbidity, ADHD with comorbidity mainly showed greater discounting of future rewards but not more negative emotions about delay. In fact, except for adults with ADHD, some studies have shown that people with schizophrenia and those with depression or more severe depressive symptoms also exhibit higher rates of delay discounting than healthy controls [63–66]. Most of the participants with ADHD in this study had comorbid major depressive disorder, so the current result may mean that ADHD comorbid with other mental disorders that may cause greater delay discounting, which will aggravate the depreciation of future rewards. Based on the comparison of the differences in reward-delay impulsivity levels between adults with ADHD and healthy controls and between different subgroups, the results of this part generally support the clinical sensitivity of the C-QDQ.

In summary, compared with the laboratory tasks of measuring delay-related difficulties, the QDQ is more more convenient to conduct. Adult ADHD has been understudied, in part because of a lack of relevant research instruments. In this study, the C-QDQ was found to have good reliability and validity. It can distinguish adults with ADHD who exhibit severe delay-related difficulties from healthy controls. In addition, the present study confirmed the existence of reward-delay impulsivity in adults with ADHD in a relatively large sample, which highlights the importance of devoting attention to delay-related behaviours of adults with ADHD. The C-QDQ is expected to promote the research on delay-related behaviours in Chinese adults with ADHD.

### Limitations and future directions

The present study has several limitations. First, ADHD is a neurodevelopmental disorder, and the symptoms of some adults with ADHD continue to old age [13]. The young age of the samples in this study have affected the representativeness of the samples, as did high level of education and the absence of the ADHD-Hyperactivity/Impulsivity subtype. Second, due to the limitations of available tasks, the two tasks in this study may not have captured only the negative emotions related to delay but rather those mixed with a variety of other factors, it may lead to a decrease in the persuasiveness of the result of the convergent validity of the delay aversion subscale. In the future, a scale assessing impatience due to waiting can be added, similar to the setting of Scheres et al. [62]. Mood changes due to delays before and after the experiment were collected separately to supplement the laboratory task and further explore the convergent validity of the delay aversion subscale. Third, due to the relatively small sample size of healthy controls, this study included all samples in the calculation of construct validity, which still may affect the use of the scale in specific populations. In the future, the sample size will be expanded to cover adults of a variety of ages and verify the reliability and validity of the scale in healthy controls and ADHD populations separately, making the research results more representative and persuasive.

## Conclusion

In conclusion, our findings demonstrate that the C-QDQ has good reliability and validity, and the ADHD group and all subgroups had higher levels of reward-delay impulsivity. The C-QDQ is a reliable and valid instrument for the assessment of delay-related behaviours in Chinese adults.

### Electronic supplementary material

Below is the link to the electronic supplementary material.

Supplementary Material 1

## Data Availability

The datasets generated or analyzed during the study are available from the corresponding author on reasonable request.

## Abbreviations

QDQ: Quick Delay Questionnaire
C-QDQ: Chinese version of the Quick Delay Questionnaire
ADHD: Attention-deficit/hyperactivity disorder
ADHD-I: ADHD-predominantly inattention type
ADHD-C: ADHD-combined type
HCs: Healthy controls
BIS-11: Barratt Impulsiveness Scale-11
TCIP: Two-Choice Impulsivity Paradigm
DDT: Delay Discounting Task
CFA: Confirmatory factor analysis
ICCs: Intraclass correlation coefficients
CVI: Content validity index
I-CVI: Item-level content validity
S-CVI/AVE: Average scale-level content validity
χ2/df: Ratio of chi-square and degrees of freedom
RMSEA: Root mean square error of approximation
SRMR: Standardized root mean squared residual
CFI: Comparative fit index
IFI: Incremental fit index
TLI: Tucker‒Lewis index
