# Supplementary material for: Psychometric properties of the Chinese version of the Quick Delay Questionnaire (C-QDQ) and ecological characteristics of reward-delay impulsivity of adults with ADHD
Source: BMC Psychiatry. 2024 Apr 2;24:251. doi: 10.1186/s12888-024-05706-2 (PMC10988885; doi:10.1186/s12888-024-05706-2)
Supplement: Supplementary file 1 — Supplementary Material 1 [file 12888_2024_5706_MOESM1_ESM.docx]

1. The results of normality tests for C-QDQ scores, age and first years of education in **healthy controls** (group 1) are as follows:

(1). Normality test of C-QDQ total scores (group 1):


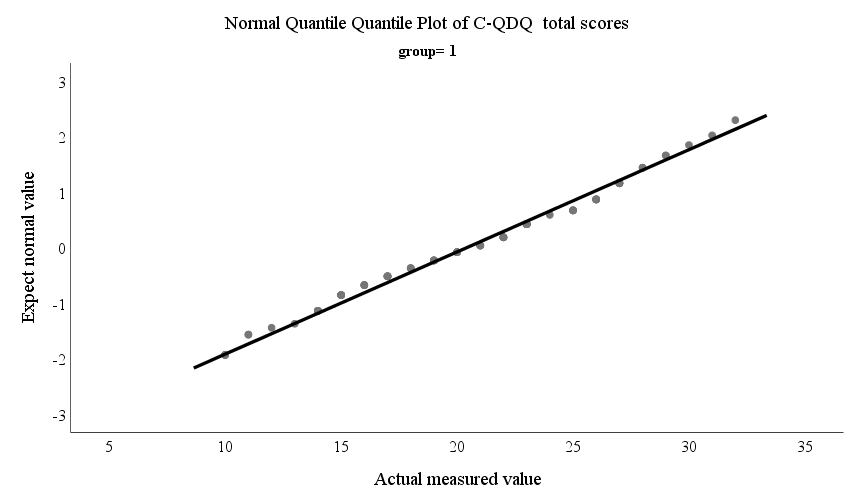


(2). Normality test of C-QDQ delay aversion subscale score (group 1):


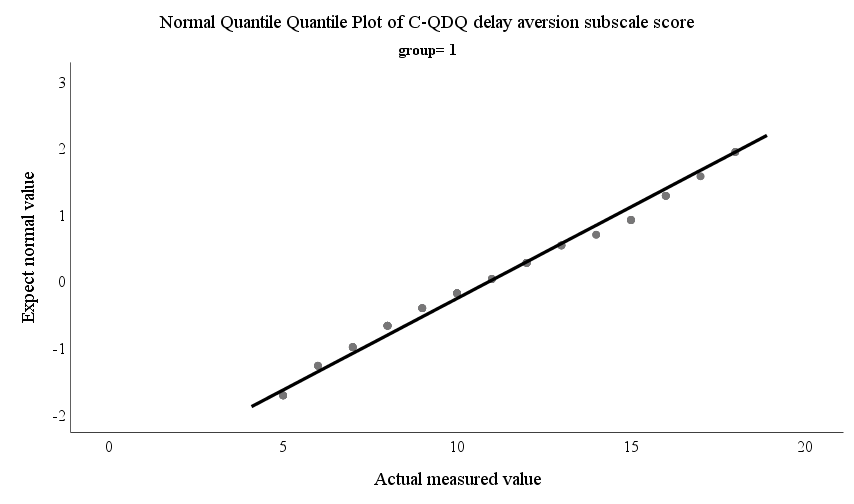


(3). Normality test of C-QDQ delay discounting subscale score (group 1):


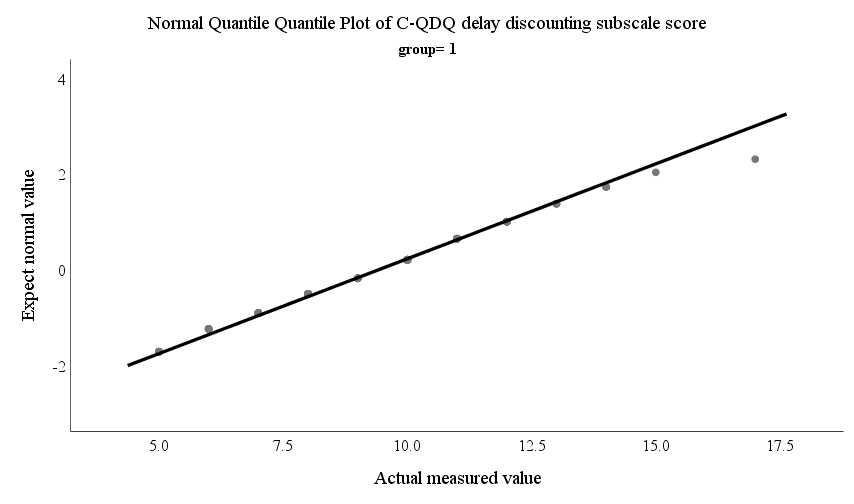


(4). Normality test of age of healthy controls (group 1):


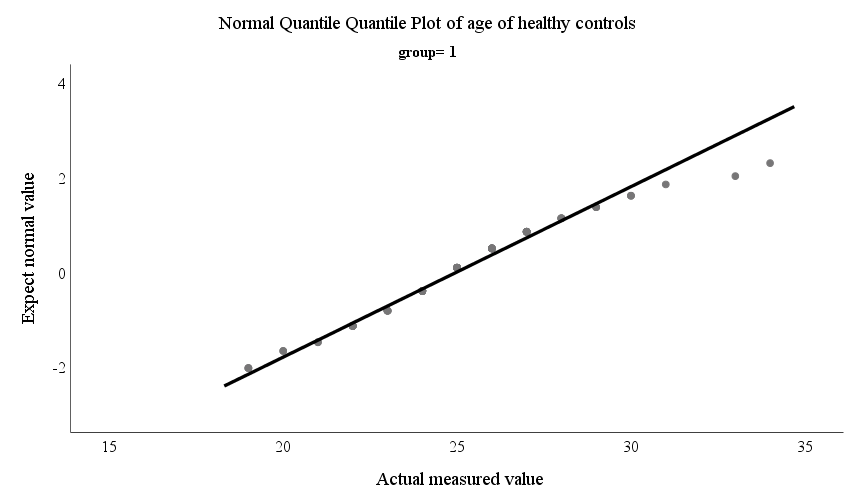


(5). Normality test for years of education of healthy controls (group 1):


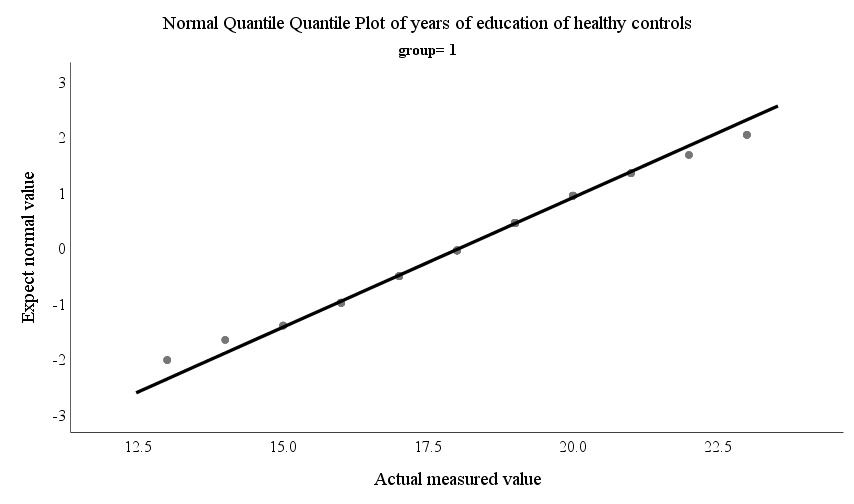


2. The results of normality tests for C-QDQ scores, age and first years of education in **adults with ADHD** (group 2) are as follows:

(1). Normality test of C-QDQ total scores (group 2):


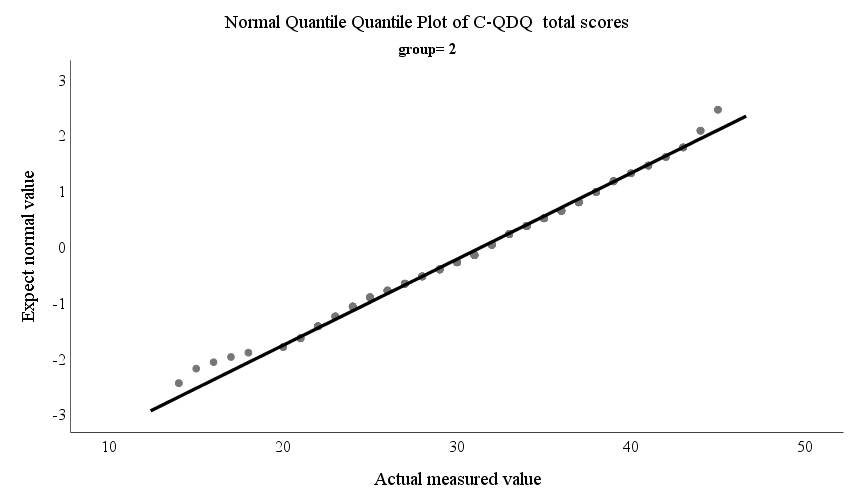


(2). Normality test of C-QDQ delay aversion subscale score (group 2):


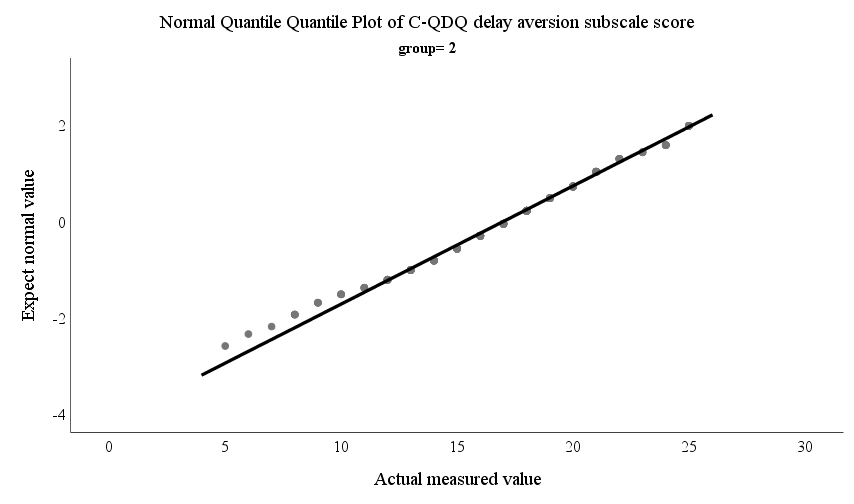


(3). Normality test of C-QDQ delay discounting subscale score (group 2):


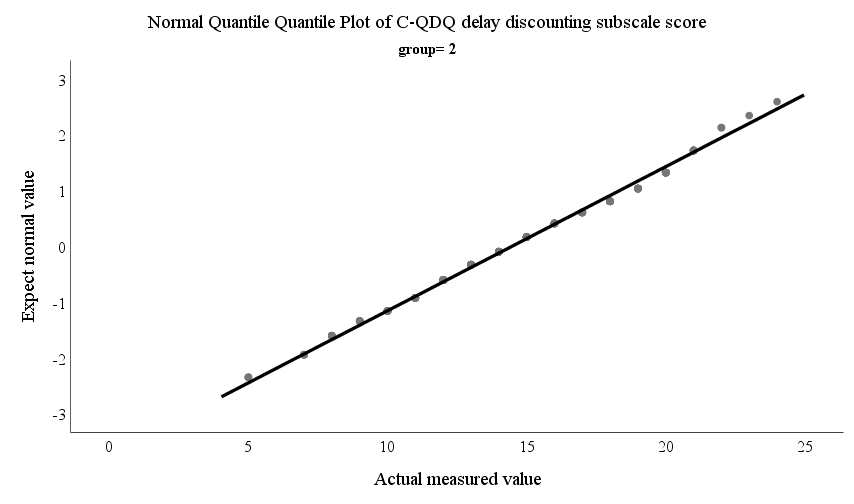


(4). Normality test of age of adults with ADHD (group 2):


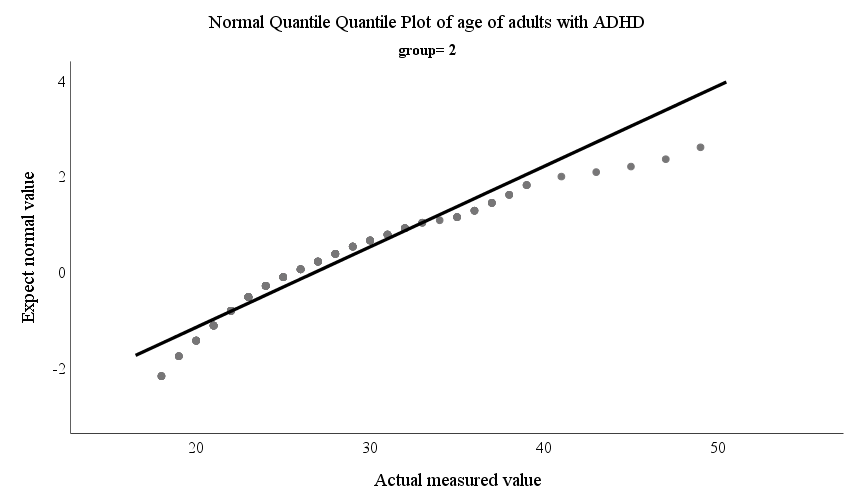


(5). Normality test for years of education of adults with ADHD (group 2):


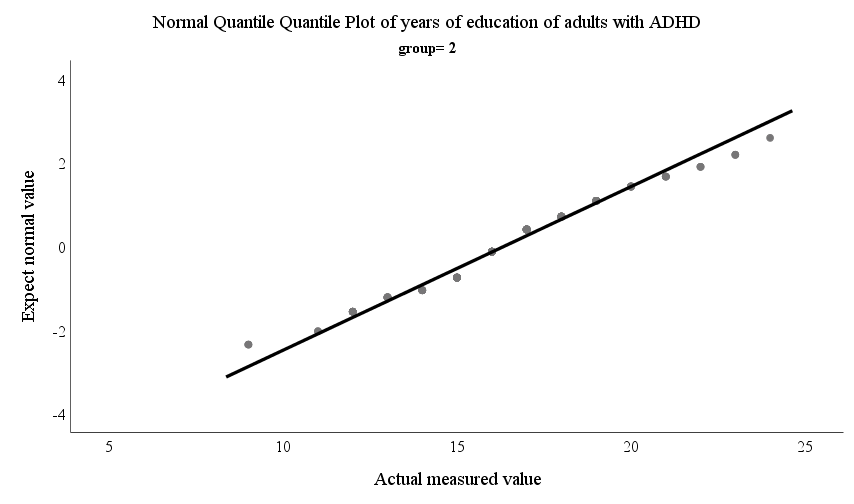


**Table S1.** The correlations between C-QDQ and BIS-11/DDT/TCIP in healthy controls (n=93).

|  | C-QDQ | |
| --- | --- | --- |
|  | Delay aversion | Delay discounting |
| BIS-11 | 0.306** | 0.324** |
| DDT^#^ | -0.088 | 0.056 |
| TCIP^^^ | 0.130 | 0.160 |

Note: ^#^=87; ^=86; **p<0.01; ***p<0.001. C-QDQ: the Chinese version of the Quick Delay Questionnaire; BIS-11: Barratt impulsiveness scale-11; DDT: delay discounting rate in delay discounting task; TCIP:the percentage of smaller sooner reward in two-choice impulsivity paradigm.

**Table S2.** The correlations between C-QDQ and BIS-11/DDT/TCIP in ADHD (n=209).

|  | C-QDQ | |
| --- | --- | --- |
|  | Delay aversion | Delay discounting |
| BIS-11 | 0.371*** | 0.516*** |
| DDT^#^ | -0.004 | -0.072 |
| TCIP^^^ | 0.223* | 0.450*** |

Note: ^#^=105; ^=86; *<0.05; **p<0.01; ***p<0.001. C-QDQ: the Chinese version of the Quick Delay Questionnaire; BIS-11: Barratt impulsiveness scale-11; DDT: delay discounting rate in delay discounting task; TCIP:the percentage of smaller sooner reward in two-choice impulsivity paradigm.
